# Removal of nonimpacted third molars alters the periodontal condition of their neighbors clinically, immunologically, and microbiologically

**DOI:** 10.1038/s41368-020-00108-y

**Published:** 2021-02-07

**Authors:** Yi Tian, Lijuan Sun, Honglei Qu, Yang Yang, Faming Chen

**Affiliations:** National Clinical Research Center for Oral Diseases, Department of Periodontology, School of Stomatology, Fourth Military Medical University, Xi’an, China

**Keywords:** Periodontitis, Third molar removal

## Abstract

Considering the adverse effects of nonimpacted third molars (N-M3s) on the periodontal health of adjacent second molars (M2s), the removal of N-M3s may be beneficial to the periodontal health of their neighbors. This study aimed to investigate the clinical, immunological, and microbiological changes of the periodontal condition around M2s following removal of neighboring N-M3s across a 6-month period. Subjects with at least one quadrant containing an intact first molar (M1), M2, and N-M3 were screened and those who met the inclusion criteria and decided to receive N-M3 extraction were recruited in the following investigation. M2 periodontal condition was interrogated before M3 extraction (baseline) and at 3 and 6 months postoperatively. Improvements in clinical periodontal indexes of M2s in response to their adjacent N-M3 removal, along with changes in inflammatory biomarkers among gingival crevicular fluid (GCF) and the composition of subgingival plaque collected from the distal sites of the M2s of the targeted quadrant were parallelly analyzed. Complete data of 26 tooth extraction patients across the follow-up period were successfully obtained and subsequently applied for statistical analysis. Compared to the baseline, the periodontal condition of M2s was significantly changed 6 months after N-M3 removal; specifically, the probing depth of M2s significantly reduced (*P* < 0.001), the matrix metalloproteinase (MMP)-8 concentration involved in GCF significantly decreased (*P* = 0.025), and the abundance of the pathogenic genera *unidentified Prevotellaceae* and *Streptococcus* significantly decreased (*P* < 0.001 and *P* = 0.009, respectively). We concluded that N-M3 removal was associated with superior clinical indexes, decreased GCF inflammatory biomarkers, and reduced pathogenic microbiome distribution within the subgingival plaque. Although the retention or removal of N-M3s continues to be controversial, our findings provide additional evidence that medical decisions should be made as early as possible or at least before the neighboring teeth are irretrievably damaged.

## Introduction

Third molar (M3) related diseases (e.g., pericoronitis, caries, odontogenic cysts, and tumors), especially caries, root resorption, and periodontal diseases of their adjacent teeth, have received extensive attention from patients and dentists.^1,2^ Although M3-associated diseases are more common in the clinic when M3s are in an impacted positioning (impacted M3s, I-M3s),^3,4^ recent evidence suggests that even nonimpacted M3s (N-M3s) may cause severe periodontal damage to their adjacent second molars (M2s).^3,5,6^

In this context, a longitudinal study over 25 years found that in healthy American males, the retention of M3s, even if they were asymptomatic, increased the risk of periodontal destruction of adjacent M2s compared to that of those without M3s.^3^ Clearly, N-M3s had not been excluded from the investigation in this study. Regarding the adverse effects of N-M3s, a German investigation reported that in the total population, M2s had a 1.45-fold odds ratio of increased probing depth (PD) in the presence of adjacent N-M3s compared to the absence of M3s.^5^ Similar conclusions based on clinical studies were also reported in Chinese patients.^7–9^ Based on orthopantomograms of 1 958 patients, our previous investigation also found that M2s with neighboring N-M3s faced a 1.77-fold increased risk of alveolar bone resorption when compared to those without adjacent M3s.^6^ Interrogating the negative influences of N-M3s from another angle, researchers also demonstrated that the removal of M3s including but not limited to N-M3s led to improved periodontal conditions for their neighboring M2s.^10,11^

The investigations mentioned above suggest that the effects of N-M3 retention on their neighboring teeth should receive more attention from both patients and dentists. Considering the difficulty of treating irreversible periodontal destruction, such as alveolar bone resorption, preventing the occurrence of periodontitis is of key importance. Since periodontal diseases were initiated by the colonization of subgingival periodontal pathogens and corresponding changes in the inflammatory-immune system,^12^ the negative impacts of N-M3s on their neighbors are likely to result from the interplay between the subgingival microbiome and host response across the local microenvironment of the periodontium. In this regard, it is reasonable to speculate that the removal of N-M3s should be able to positively alter the local immunological and microbiological conditions around their adjacent M2s.

In this study, the clinical, immunological, and microbiological changes in the periodontal condition around M2s in terms of clinical periodontal indexes, inflammatory biomarkers in gingival crevicular fluid (GCF), and the composition of subgingival plaque of M2s were investigated following removal of their neighboring N-M3s across a 6-month period. The specific aim of the study was to identify how N-M3 removal alters the periodontal condition of their neighbors and to provide additional evidence that N-M3s lead to adverse effects on the periodontal health of adjacent M2s.

## Results

### Subjects enrolled

**Fig. 1:**
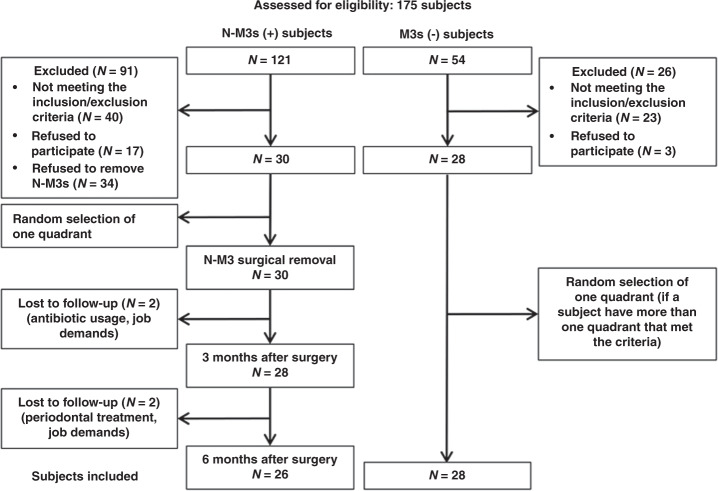
Flow chart of the study design. *N*, number of patients; N-M3s (+), second molars (M2s) with neighboring nonimpacted third molars; M3s (−), M2s without neighboring third molars

From May 2019 to June 2019, 121 subjects were recorded in the N-M3s (+) group and 54 in the M3s (−) group at the prescreening stage. Based on inclusion and exclusion criteria and/or the agreement of tooth extraction, only 30 of the N-M3s (+) and 26 of the M3s (−) subjects were included in the longitudinal study (with one included quadrant per patient, randomly selected if multiple quadrants were eligible); 4 participants were lost or excluded during the 6-month longitudinal observation stage. After a 6-month follow-up, complete data were successfully obtained from 26 participants in the N-M3s (+) group and 28 participants in the M3s (−) group, and those data were applied for the subsequent statistical analysis (Fig. 1).

### Sociodemographic information and clinical periodontal indexes of the participants

The demographic characteristics and locations of target quadrants of the 26 N-M3s (+) and 28 M3s (−) participants were homogeneous between groups (*P* > 0.05, Table 1). Clinical periodontal indexes of the targeted M2s obtained at the baseline (T0) and across the follow-up period (3 months postoperatively was recorded as T1, while 6 months postoperatively was recorded as T2) are presented in Table 2. Compared to the baseline measurement (T0) in the N-M3s (+) group, PD was significantly reduced 6 months (T2) after the removal of the neighboring N-M3s (*P* < 0.001), while changes in the plaque index (PLI), the prevalence of at least three sites that bled after probing (BOP+), at least one probing site ≥5 mm (PD5+), and GCF volume showed no statistical significance among T0–T2 (*P* = 0.280, *P* = 0.307, *P* = 0.247, and *P* = 0.857, respectively). When the M3s (−) group was used as the control, significantly increased PD, an elevated prevalence of BOP+, and significantly increased GCF volume were recorded before N-M3 extraction (T0) (*P* < 0.001, *P* = 0.006, and *P* = 0.022, respectively), but only PD reached a similar level to that of the control 6 months postoperatively (T2) (*P* = 0.685), while the prevalence of BOP+ and the GCF volume remained greater than those of the control (*P* = 0.012 and *P* = 0.035, respectively). To identify the potential influence of N-M3s on tissue damage of their neighbors, we compared the mean PD of distobuccal and distolingual sites (dPD) for first molars (M1s) and M2s of the same quadrant across the follow-up stage (Table 3). As expected, M2s had a greater dPD than that of M1s was examined when N-M3s was present (*P* < 0.001). Compared to the value at T0, the removal of N-M3s led to a significant reduction in dPD for M2s at both the T1 and T2 time points (*P* = 0.001 and *P* < 0.001, respectively), but no significant change was found in the dPD for M1s (*P* > 0.05). Again, the dPD for M2s 6 months postoperatively (T2) was similar to that of M2s in the M3s (−) group (*P* = 0.956).

**Table 1:** Demographic characteristics of participants (*N* = 54) and locations of targeted quadrants

Characteristics	All (*N* = 54)	N-M3s (+) (*N* = 26)	M3s (−) (*N* = 28)	*P*
*Age/years*				
Mean ± SD	25.9 ± 7.4	24.9 ± 6.2	26.8 ± 8.4	0.361
Range	18–56	18–48	18–56	
*Gender/%*				
Male	53.7	40.7	60.7	0.284
Female	46.3	59.3	39.3	
*Ethnicity/%*				
Han Chinese	98.1	96.3	100.0	0.481
Other	1.9	3.7	0.0	
*Education level/%*				
College or above	83.3	88.5	78.6	0.470
High school or below	16.7	11.5	21.4	
*Dental scaling frequency/%*				
At least once every 2 years	24.1	23.1	25.0	0.869
Never	75.9	76.9	75.0	
*Dental visit frequency/%*				
At least once every 2 years	37.0	35.7	35.7	0.700
Only when necessary	63.0	64.3	64.3	
*Locations of targeted quadrants/%*				
Maxillary	87.0	96.2	78.6	0.102
Mandibular	13.0	3.8	21.4	

*Note*: N-M3s (+), M2s with neighboring nonimpacted third molars; M3s (−), M2s without neighboring third molars; SD standard deviation.

**Table 2:** Clinical periodontal changes in M2s in participants with neighboring N-M3s (*N* = 26) in response to N-M3 removal and clinical periodontal indexes of M2s in participants without neighboring M3s (*N* = 28)

Groups		Teeth (M2s)	PD/mm (mean ± SD)	PLI (mean ± SD)	PD5+/%	BOP+/%	GCF/µL (mean ± SD)
N-M3s (+)	T0	26	3.01 ± 0.45	2.17 ± 0.62	15.4	38.5	1.25 ± 0.45
	*P*^a^		<0.001	0.150	0.184	0.006	0.022
	T1	26	2.53 ± 0.32	1.88 ± 0.59	3.8	23.1	1.23 ± 0.37
	*P*^*a*^		0.390	0.812	0.100	0.135	0.023
	*P*^1^		<0.001	NS	NS	NS	NS
	T2	26	2.48 ± 0.23	1.98 ± 0.54	7.7	34.6	1.23 ± 0.46
	*P*^a^		0.685	0.948	0.604	0.012	0.035
	*P*^2^		<0.001	NS	NS	NS	NS
M3s (−)		28	2.49 ± 0.30	1.95 ± 0.50	3.6	7.1	0.97 ± 0.37

*Note*: M2s, second molars; N-M3s, nonimpacted third molars; M3s, third molars; N-M3s (+), M2s with neighboring N-M3s; M3s (−), M2s without neighboring M3s.

PD, probing depth; PLI, plaque index; PD5+, at least one probing site with PD ≥ 5 mm; BOP+, sites of bleeding on probing ≥ 3; GCF, gingival crevicular fluid.

T0, before N-M3 removal; T1, 3 months after N-M3 removal; T2, 6 months after N-M3 removal.

SD, standard deviation; *P*^a^, compared between N-M3s (−) and N-M3 (+) (T0, T1, or T2); *P*^1^, compared between T0 and T1; *P*^2^, compared between T0 and T2; *P*^1^ and *P*^2^ values were adjusted for multiple comparisons by the Bonferroni correction; NS, no significance; because the PLI, the prevalence of BOP+ and PD5+, and the GCF volume showed no significant change among T0–T2, pairwise comparisons were not conducted between T0 and T1 (or T0 and T2); the significance value was set at 0.05.

**Table 3:** Changes in the dPD of M1s and M2s in participants with neighboring N-M3s (*N* = 26) in response to N-M3 removal and clinical periodontal indexes of M1s and M2s in participants without neighboring M3s (*N* = 28)

Groups		Teeth	dPD/mm (mean ± SD)		*P*^b^
		(M2s)	M1s	M2s	
N-M3s (+)	T0	26	2.69 ± 0.47	3.65 ± 0.85	<0.001
	*P*^a^		0.598	<0.001	
	T1	26	2.58 ± 0.52	2.83 ± 0.51	0.074
	*P*^*a*^		0.242	0.053	
	*P*^1^		NS	<0.001	
	T2	26	2.54 ± 0.37	2.54 ± 0.51	0.888
	*P*^a^		0.043	0.956	
	*P*^2^		NS	<0.001	
M3s (−)		28	2.73 ± 0.40	2.55 ± 0.39	0.040

*Note*: M2s, second molars; N-M3s, nonimpacted third molars; M3s, third molars; N-M3s (+), M2s with neighboring N-M3s; M3s (−), M2s without neighboring M3s.

dPD, mean probing depth of distobuccal and distolingual sites.

T0, baseline; T1, 3 months after M3 extraction; T2, 6 months after M3 extraction.

SD, standard deviation; *P*^a^, compared between N-M3s (−) and N-M3 (+) (T0, T1, or T2); *P*^b^, compared within the same period; *P*^1^, compared between T0 and T1; *P*^2^, compared between T0 and T2; *P*^1^ and *P*^2^ values were adjusted for multiple comparisons by the Bonferroni correction; NS, no significance; because the dPD showed no significant change among T0–T2, pairwise comparisons were not conducted between T0 and T1 (or T0 and T2); the significance value was set at 0.05.

### Inflammatory biomarkers in GCF

**Fig. 2:**
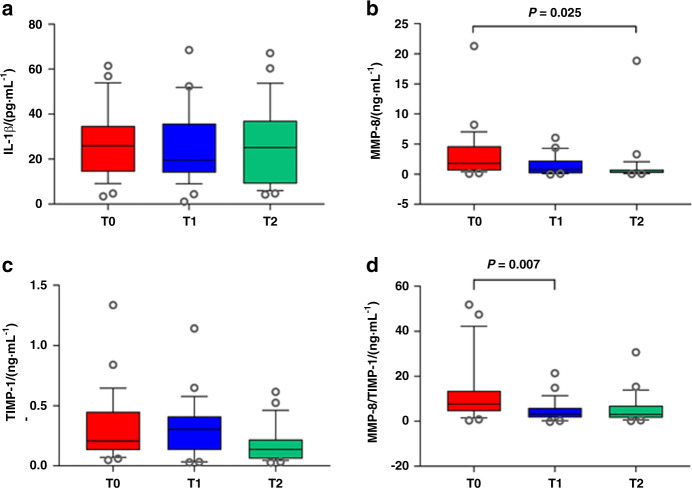
Changes in inflammatory biomarkers in the GCF of M2s in participants with neighboring N-M3s (*N* = 26) in response to N-M3 removal. Tukey box plots depict the concentrations of **a** IL-1β, **b** MMP-8, **c** TIMP-1, and **d** the ratio of MMP-8/TIMP-1 involved in GCF. Open circles indicate “outlier” values. *P*, compared between different periods, analyzed by the Friedman test, and were adjusted for multiple comparisons by the Bonferroni correction; T0, before N-M3 removal; T1, 3 months after N-M3 removal; T2, 6 months after N-M3 removal

Considering that the concentrations of specific inflammatory biomarkers involved in GCF can reflect the local periodontal immune condition of a tooth, the concentrations of interleukin (IL)-1β, matrix metalloproteinase (MMP)-8, and tissue inhibitors of matrix metalloproteinase (TIMP)-1 were measured in the GCF samples obtained at the baseline (T0) and across the follow-up period (T1 and T2) to monitor the immunological changes in the periodontal condition of M2s in response to N-M3 removal (Fig. 2). Although the removal of N-M3s resulted in no significant changes in IL-1β concentration (Fig. 2a), it was found that, following N-M3 extraction, the concentration of MMP-8 decreased with time (from T0 and T1 to T2), with a significant change in MMP-8 concentration was found at T2 (compared to the value at T0, *P* = 0.015, Fig. 2b). The TIMP-1 concentration at T2 was also reduced but was not significantly different from the value at T0 (no statistical significance, *P* > 0.05, Fig. 2c). Importantly, the MMP-8/TIMP-1 ratio was reduced following N-M3 extraction, and it was found to be significantly lower at T1 than that at T0 (*P* = 0.006, Fig. 2d), while the reduction in the MMP-8/TIMP-1 ratio showed no statistical significance at T2 (*P* > 0.05).

### Microbial composition in subgingival plaque

**Fig. 3:**
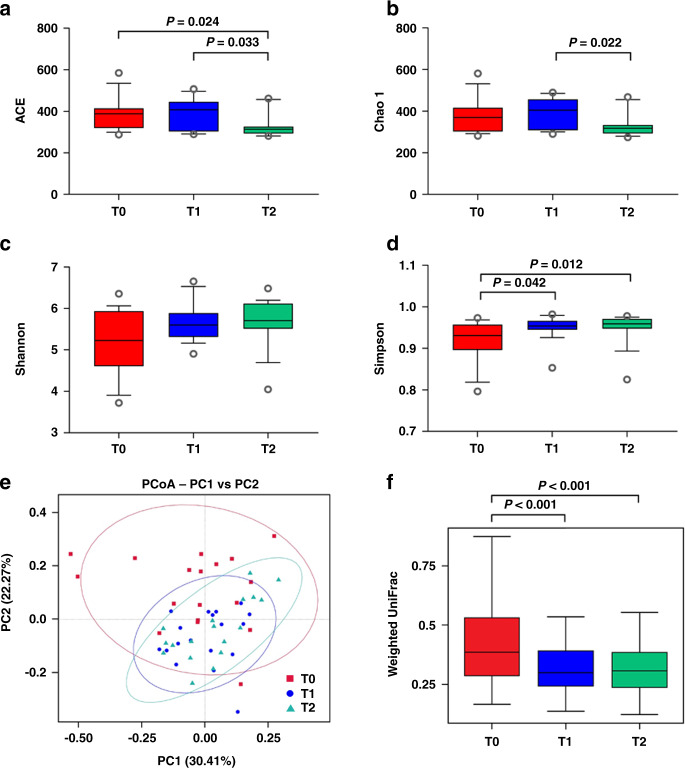
Changes in the microbial diversity of the subgingival plaque of M2s in participants with neighboring N-M3s (*N* = 18) in response to N-M3 removal. Community richness was evaluated by **a** ACE and **b** Chao 1, while diversity was analyzed by **c** Shannon and **d** Simpson indexes. **e** Principal coordinate analysis (PCoA) plot based on weighted UniFrac distances and **f** weighted UniFrac distances depict beta diversity. T0, before N-M3 removal; T1, 3 months after N-M3 removal; T2, 6 months after N-M3 removal

**Fig. 4:**
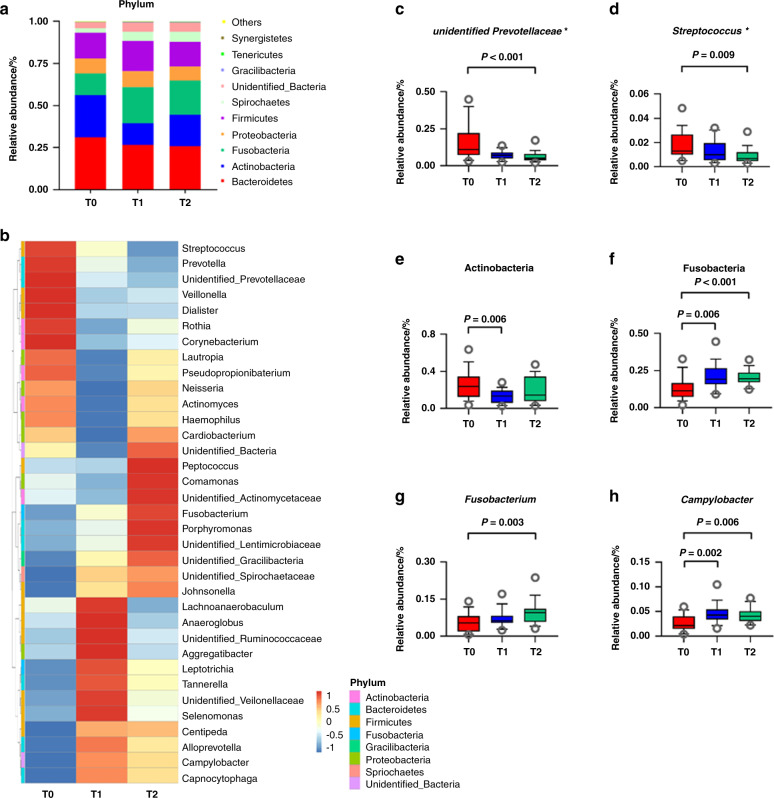
Changes in the microbial composition of subgingival plaque of M2s in participants with neighboring N-M3s (*N* = 18) in response to N-M3 removal. **a** Top ten relative abundance of bacterial phyla distributed within the subgingival plaque. **b** Heatmap shows the relative abundance of the top 35 genera distributed within the subgingival plaque. Colors reflect the abundance of genera from high (*red*) to low (*blue*). **c**, **d** The changes in the genera *unidentified Prevotellaceae* and *Streptococcus*, presented by Tukey box plots. **e**–**h** The changes in the phyla Actinobacteria and Fusobacteria as well as genera *Fusobacterium* and *Campylobacter*, presented by Tukey box plots. T0, before N-M3 removal; T1, 3 months after N-M3 removal; T2, 6 months after N-M3 removal; *, putative periodontopathic genera

Considering that the periodontal diseases are initiated by the colonization of subgingival periodontal pathogens, the microbial diversity and composition of the subgingival plaque samples obtained at the baseline (T0) and across the follow-up period (T1 and T2) were determined to monitor the microbiological changes in M2 periodontal condition in response to N-M3 removal (Figs. 3 and 4). Among the 26 participants who underwent tooth extraction, subgingival plaque samples from 8 participants were unqualified; therefore, samples from only 18 participants (obtained at T0–T2) were analyzed using high-throughput sequencing. Finally, a total of 4 492 092 high-quality sequences (mean per sample: 83 167 ± 7 220; range per sample: 5 069 to 93 753) were generated, and 19 194 operational taxonomical units (OTUs) were obtained using a 97% similarity level.

After the removal of N-M3s, the microbial richness in the subgingival plaque of adjacent M2s increased and then decreased (Fig. 3a, b). Compared with T0, the ACE analysis showed that the microbial richness was significantly reduced at T2 (*P* = 0.024, Fig. 3a). The microbial diversity increased after N-M3 removal (Fig. 3c, d), and compared with T0, a significant increase in the Simpson index was found at T1 and T2 (*P* = 0.042 and *P* = 0.012, respectively, Fig. 3d). Moreover, the variations in the microbial community structures were clearly different between T0 and T1 (T0 and T2) presented by principal coordinate analysis (PCoA) (Fig. 3e), and significant decreases were found at T1 and T2 (*P* < 0.001 and *P* < 0.001, respectively, Fig. 3f).

The microbial composition of the subgingival plaque samples of M2s in response to N-M3 removal was further analyzed (Fig. 4). At the phylum level, Bacteroidetes, Actinobacteria, Fusobacteria, Proteobacteria, Firmicutes, and Spirochetes were the dominant phyla, accounting for more than 90% of the sequences (Fig. 4a). At the genus level, the distributions of the top 35 genera at T0–T2 were different in the heatmap (Fig. 4b). In addition, pathogenic genera such as *unidentified Prevotellaceae* and *Streptococcus* were significantly decreased at T2 compared to T0 (*P* < 0.001 and *P* = 0.009, respectively, Fig. 4c, d). Compared to T0, the relative abundance of Actinobacteria was decreased at T1 and T2 (*P* = 0.006 and *P* > 0.05, Fig. 4e), while Fusobacteria, *Fusobacterium*, and *Campylobacter* were significantly increased at T2 (*P* < 0.001, *P* = 0.003, and *P* = 0.006, respectively, Fig. 4f–h).

## Discussion

In recent years, the negative impact of N-M3s, as a common clinical problem (Supplementary Fig. 1), has attracted increasing attention from dentists.^5,6^ However, there is limited evidence from the laboratory-based assessments of the periodontal risks associated with N-M3s. We also conducted a pairwise comparison of the periodontal condition of bilaterally matched quadrants of M2s with neighboring N-M3s or without M3s from the enrolled participants and found that the presence of N-M3s was associated with increased PD and dPD of neighboring M2s and an increased abundance of pathogenic microbiomes within subgingival plaque compared to the absence of M3s (data not shown). Thus, in this 6-month longitudinal study, we investigated the clinical, immunological, and microbiological changes in the periodontal condition around M2s following removal of neighboring N-M3s. We found that N-M3 removal was associated with an improvement of periodontal condition around neighboring M2s, including superior clinical indexes, decreased GCF inflammatory biomarkers, and reduced pathogenic microbiome distribution within the subgingival plaque.

Regarding clinical periodontal indexes, our data support the findings of previous studies, showing beneficial changes in variables such as PD and dPD for M2s in response to neighboring M3 removal.^10,13,14^ However, the reduction in PLI and the prevalence of PD5+ in the present study did not reach statistical significance (Table 2), which may be due to the strict inclusion/exclusion criteria, and the fact that most of the M3s in our study were located in maxillary (87%) while previous work focused on mandibular M3s. All subjects who came to our school for oral hygiene maintenance purposes during the investigation period were screened according to inclusion/exclusion criteria and their consent to have the N-M3s removed (Fig. 1). The higher proportion of targeted quadrants located in the maxillary supported our previous cross-sectional studies of N-M3s.^6,8,11^ Both radiological and clinical investigations showed that there were more N-M3s in the maxilla, while I-M3s were more likely to be in the mandible.^5,9^ The different influences of N-M3s in maxilla and mandible have been discussed previously. We found that, compared to maxillary N-M3s, N-M3s located in the mandible showed greater risks of periodontal pathology in neighboring M2s.^6,8,9,11^ Separate analysis of the targeted maxillary and mandibular quadrants would be optimal, however, since there was only one mandibular targeted quadrant that underwent N-M3 extraction, this analysis was not conducted. In the present study, the degree of clinical improvement of PD and dPD for M2s was further compared with control subjects lacking M3 (Tables 2 and 3). The reductions in PD and dPD after N-M3 removal reached a level similar to that of the control subjects. Interestingly, no significant changes in PD5+ prevalence or GCF volume were found postoperatively, while BOP+ prevalence and GCF volume were greater than those of the control. These results may partly explain the relatively low percentage of periodontal healing of M2s with deep PD (PD ≥ 5 mm) observed by Sun et al.^11^ In this region around M3s, where accumulated microbial biofilms are difficult to be completely eradicated by mechanical methods,^15^ periodontal pathogens colonize readily and are capable of stimulating inflammatory and immune activities.^16–18^ As a result, patients are often at risk for periodontal diseases in the M3 region, and the presence of M3s may cause periodontal damage to their neighboring M2s.

In addition to the analysis of GCF volume, GCF is widely used to investigate the periodontal inflammatory biomarkers because of its close proximity to the periodontium. Studies have identified multiple biomarkers in the GCF during the inflammatory process of periodontal diseases and treatments.^19,20^ Among those biomarkers, IL-1β and MMP-8 have shown diagnostic potential for periodontitis.^21–23^

IL-1β participates in alveolar bone resorption and is expected to be significantly downregulated after periodontal treatments.^19,24^ It was demonstrated that an increased concentration of IL-1β was present in GCF if PD5+ was detected around M3s compared with patients with PD less than 5 mm.^18^ In the current investigation, we did not detect significant differences in IL-1β concentration (Fig. 2a). This could be explained by the lack of a significant reduction in the prevalence of PD5+ in M2s in response to N-M3 removal.

MMP-8 (or collagenase 2), as a prominent collagenase, is involved in the degradation of the extracellular matrix and basement membrane.^25–27^ MMPs are controlled and inhibited by specific tissue inhibitors, mainly TIMP-1, and a shift in the balance between MMP-8 and TIMP-1 can lead to the progression of periodontal diseases.^28,29^ In this study, our results showed that the MMP-8 concentration and the MMP-8/TIMP-1 ratio in GCF decreased following N-M3 extraction: MMP-8 concentration was significantly reduced at T2, and the MMP-8/TIMP-1 ratio was significantly reduced at T1 (Fig. 2b, d). As no other studies have explored concentrations of MMP-8 and TIMP-1 around M2s, the results of the present study cannot be compared with those in the literature. However, these reductions were associated with tissue reattachment in the healing process and were in agreement with earlier studies showing that clinical indexes improved with decreased concentrations of MMP-8 after periodontal treatments.^30,31^ The concentration of TIMP-1 has been reported in the literature to increase or decrease after various treatments,^32,33^ indicating that the regulation of TIMP-1 may not be solely dependent on MMPs. The observations of the MMP-8/TIMP-1 ratio from our study were consistent with previous investigations. A higher MMP-8/TIMP-1 ratio was detected in patients with periodontal inflammation,^34,35^ and it was positively associated with the periodontal inflammatory burden index.^36^ In a paper by Teixeira et al.^37^, MMP-8 concentration and MMP-8/TIMP-1 ratio were significantly higher in patients with PD ≥ 6 mm. In addition to local inflammatory status, several studies suggested that M3s were associated with higher systemic inflammation than controls,^38^ and oxidative stress biomarkers were reduced significantly after M3 extraction.^39^

The microbiological findings showed that M3 extraction effectively reduced microbial richness and increased microbial diversity following N-M3 removal. The relatively high diversity, species homogeneity, and sample-to-sample similarity observed in T2 implied a more complex community after extraction, thus being healthier and more stable (Fig. 3).^40,41^ In other words, before N-M3 extraction, there were more microbes with low relative abundance at baseline. Moreover, the results of PCoA revealed that the microbial community structures significantly changed after M3 extraction, showing greater concentration in samples at T2 compared to baseline. The relationship among the microbial communities in different samples based on the unweighted pair group method with arithmetic mean (UPGMA) clustering analysis also suggested that samples at baseline could be easily discriminated from the samples at T1 and T2 (Supplementary Fig. 2). This suggested that the microbial community might have undergone a similar healing process postoperatively. These data are in accordance with Shen et al. who observed greater similarity of microbial composition in patients without adverse reactions following M3 extraction, compared with patients diagnosed with dry socket.^42^ Our data were also in accordance with previous investigators who found remaining Shannon diversity values at pretreatment levels up to 6 months after extraction.^43^ However, the increased Simpson index after M3 extraction could be due to the continuous outflow of GCF from the gingival sulcus, which could protect the subgingival microenvironment. In addition, the subgingival microbiome is site-specific and is mainly influenced by the periodontal depth and local inflammation.^44^

In the current study, the relative abundance of Fusobacteria was significantly altered among time points. Fusobacteria belongs to late colonizers of the healthy core oral microbiome,^45,46^ and they significantly increased in response to N-M3 removal. At the genus level, *unidentified Prevotellaceae* and *Streptococcus* significantly decreased 6 months postoperatively. Some *Prevotella* and *Streptococcus* species have been identified as putative periodontal pathogens and are closely related to the orange complex.^47–50^ Our findings indicated that *unidentified Prevotellaceae* could be associated with the onset or persistence of periodontal inflammation involved in the M3 region. The genus *Streptococcus* has been previously reported to have a significantly higher abundance of in response to nonsurgical periodontal therapy.^51,52^ In contrast, our findings showed a decreased abundance of *Streptococcus*. A possible explanation could be the relatively longer follow-up time in our study and the differences in participants. In addition, *Fusobacterium* and *Campylobacter* significantly increased at T2, and both of them have been detected in the healthy oral cavity.^45^ The correlations between the periodontopathic microbiome and inflammatory biomarkers were further analyzed by Spearman correlation (Supplementary Table). Although no statistical significance was observed, the relative abundance of *unidentified Prevotellaceae* was positively correlated with inflammatory biomarkers in GCF.

Interestingly, our findings in comparisons of bilaterally matched quadrants also showed significantly higher abundance of Fusobacteria and lower abundance of *unidentified Prevotellaceae* in the M3s (−) group (data will be published elsewhere), which was similar to patients who received N-M3 removal at the 6-month follow-up. These results indicated that the relationship between Fusobacteria and *unidentified Prevotellaceae* could have crucial roles in the negative influence of N-M3s. Previous studies also demonstrated a greater proportion of putative periodontal pathogens in the M3 region than incisor sites,^16^ and these pathogens may initiate periodontitis in young adults.^18^ In that case, protective M3 extraction would be beneficial since the extraction made it accessible for the distal surface of M2s to maintain better self-cleaning, forming a healthy periodontium, and may help prevent the development and progression of periodontitis.

We are well aware of the strengths and limitations of our study. On the one hand, this study combines the clinical, immunological, and microbiological parameters, providing comprehensive evidence on which to base future research into the impact of N-M3s on the periodontal health of neighboring teeth. On the other hand, the nonrandomized nature of the study design and the relatively short follow-up time are limitations of the study. The removal of M3s carries costs and risks, therefore, conducting a well-designed randomized (or split mouth) clinical trial is challenging. However, randomized methods were applied to select study quadrants. In addition, there were no significant differences in clinical periodontal indexes or immunological findings between 3 and 6 months postoperatively, which indicated that the periodontium of M2s was in a stable state 6 months after the extraction.

Over time, our group has conducted several studies on the negative periodontal influence of N-M3s. First, we noticed the risk of alveolar bone resorption of neighboring M2s from the radiological investigation.^6^ Then, we discovered clinical manifestations of deeper PD with the presence of adjacent N-M3s.^8,9^ In search of a clinical strategy for governing N-M3s, we demonstrated that M2s exhibited improved periodontal conditions following N-M3 removal.^11^ This study explored the immunological and microbiological reasons for the improvement of the periodontal condition around adjacent teeth after N-M3 extraction. However, it is still too early to make a conclusion based on current studies. We are currently exploring the clinical strategy and personalized treatment plan for N-M3s through population-based multicenter clinical trials. In the future, we plan to further explore the alterations of a larger array of biomarkers in other body fluids (such as blood and saliva) and verify the periodontal hazards of the pathogenic microbiome found in this article through animal experiments. Future studies on the current topic are therefore recommended, and the above limitations should be taken into consideration.

## Materials and methods

### Ethics of the study

The present cohort study fully complied with the Declaration of Helsinki on experimentation involving humans. Verbal and written information describing the nature of the study was given to all enrolled participants and signed informed consent forms were obtained prior to enrollment of the subjects in the investigation. The Ethics Committee and Institutional Review Board of School of Stomatology at the Fourth Military Medical University (FMMU) approved the protocol (ethics committee protocol number: 2017034; date of registration: 22 December 2017).

### Inclusion and exclusion criteria

This study was conducted at the School of Stomatology FMMU (Xi’an, China). Consecutive subjects with at least one quadrant having an intact M1, M2, and N-M3 were screened, and those who decided to undergo N-M3 extraction were asked to participate in this investigation. N-M3 was defined as an asymptomatic third molar that reached the occlusal plane, lacked rotation, and could be examined on all five surfaces, regardless of its functionality.^8^ The recruited subjects were assigned to the N-M3s (+) group. At the same time, subjects with at least one quadrant having intact M1 and M2 but no M3 (congenitally absent or extracted more than 6 months prior according to case history and radiography) were also recruited for the control for clinical assessment as the M3s (−) group. The subject-related inclusion criteria were as follows: (i) age of ≥18 years of age; (ii) no crown or bridge prostheses, severe caries, or advanced periodontitis (PD ≥ 7 mm and/or radiographic imaging of bone resorption more than 20% of the tooth length) in the examined quadrant^38^; and (iii) had at least 20 natural permanent teeth, with functional occlusal contact in dentistry.

Subjects were excluded if they (i) were smokers; (ii) were females who were pregnant or lactating; (iii) had any systemic condition that could influence the course of periodontal disease (e.g., diabetes, hypertension, or a history of radiation or cancer therapy); (iv) had received orthodontic treatment; (v) had undergone periodontal treatment within 6 months, received antibiotics/anti-inflammatory drugs/oral contraceptives within 3 months, used mouthwash that contained antibiotics within 3 months, or (vi) suffered acute dental infections in need of emergency treatment.

### Sample size estimation

The primary outcome of the present study was the PD change of M2s in response to neighboring N-M3 removal; hence, similar to a previous study,^11^ subject size calculation was performed according to the PD change using specialized software (G*Power version 3.0.8, Heinrich Heine University, Düsseldorf, Germany). The power calculation analysis revealed that to detect a 0.5 mm difference in PD at the 0.05 probability level with a power of 80%, the required minimum sample size for N-M3 extraction was 21. Assuming a 20% drop-out rate, a minimum of 24 subjects was suggested to be recruited.

### Recruitment of subjects

All subjects who came to our school for oral hygiene maintenance purposes during the investigation period were screened as potential participants, and those recruited to this study were based on their agreement to participate and met the inclusion/exclusion criteria. For N-M3s (+) subjects, their recruitment into the investigation was also dependent on their agreement to undergo tooth extraction at a certain stage of the study.

### Study design

In this 6-month longitudinal study, changes in clinical periodontal indexes, inflammatory biomarkers in GCF, and the composition of subgingival plaque of M2s in response to N-M3 removal were analyzed. For the N-M3s (+) group, clinical periodontal indexes and GCF and plaque samples were collected prior to the surgical procedure (baseline, T0) as well as at the follow-up stage (3 months postoperatively, T1; 6 months postoperatively, T2). Since the subject-specific microbiota was distributed in periodontally healthy individuals,^53^ immunological and microbial analyses were only performed on subjects who undergone N-M3 removal. Therefore, for the M3s (−) group, only clinical periodontal indexes were collected at baseline once the subjects were successfully enrolled in the study to determine the degree of clinical improvement.

The decision to extract N-M3s was made by experienced dentists and they were not involved in any research procedure of the present study. Besides observing the clinical, immunological, and microbiological changes before M3 extraction and postoperatively, the researchers of this study did not interfere with the decision to extract teeth. The N-M3s removal surgery was performed under local anesthesia (Perimacaine, Z.I. du Phare, Merignac, France). For all patients, soft tissue flaps, tooth section, and/or osteotomy were unnecessary, and once the tooth was removed, the socket was carefully inspected. Afterward, patients were instructed to take local disinfectant buccal tablets (2 mg; Cetylpyridinium Chloride Buccal Tablets, HWANG’S Pharmaceutical Co., Ltd., Taiwan) every 6 h for 3 days. Analgesics (60 mg; Loxoprofen Sodium Capsules, QIDU Pharmaceutical Co. Ltd., Shandong, China) were prescribed every 8 h for 3 days when necessary.^11^ Participants were automatically excluded if they (i) did not complete the follow-up, (ii) received antibiotics/anti-inflammatory drugs, or (iii) underwent periodontal, restorative, or orthodontic treatment during the follow-up period. If a participant had more than one eligible quadrant for investigation, only one quadrant was randomly selected for research.

### Sociodemographic information of the participants

All participants were asked to answer all questions on a questionnaire including age, gender, ethnicity, education level, dental scaling, and dental visit frequency. However, only those who completed the study were used for analysis.

### Clinical periodontal indexes

For all participants, an experienced periodontist recorded the PD, BOP, and PLI of the molars located in the examined quadrant; PD and BOP were examined at six sites (mesiobuccal, midbuccal, distobuccal, mesiolingual, midlingual, and distolingual) using a manual periodontal probe (Hu-Friedy, Chicago, IL, USA).^11^

PD was recorded as the mean value of the six sites. PD5+ was recorded as at least one probing site ≥5 mm. BOP+ was recorded when at least three sites of M2 showed any trace of blood after probing. The PLI of the buccal and lingual surfaces was recorded according to the scoring criteria previously described in the literature.^11^ Specifically, dPD was recorded as the mean PD of distobuccal and distolingual sites.

### GCF sampling and inflammatory biomarkers

For participants receiving tooth extraction, GCF samples were collected at T0–T2 from the distobuccal and distolingual sites of the M2s of the targeted quadrant by means of sterile PerioPaper strips (Oraflow, Smithtown, NY, USA), and performed after air-drying, isolation with cotton rolls, and removal of supragingival plaque. The paper strips were introduced into the sulcus until resistance was felt and kept in situ for 30 s. Strips were discarded when contaminated with visible blood or saliva. Subsequently, each strip was measured for fluid volume with a precalibrated Periotron 8000 (Oraflow Inc.). Then, two strips were pooled and eluted into an Eppendorf tube containing 500 μL of phosphate-buffered saline (PBS) and placed immediately into dry ice. The readings from the Periotron 8000 were converted to actual volume (μL) according to the manufacturer’s instructions. Each sample was vortexed and centrifuged at 3 000 × *g* for 10 min. Then, the paper strips were removed, and the supernatant was stored at −80 °C until the day of the analysis.^54^ Concentrations of IL-1β, MMP-8, and TIMP-1 involved in the GCF samples were determined using commercially available enzyme-linked immunosorbent assay kits from Quantikine (R&D Systems, Inc., MN, USA) according to the manufacturer’s instructions. The detection levels of the kits were 1 pg/mL for IL-1β, assay range 3.9–250 pg·mL^−1^, 0.06 ng·mL^−1^ for MMP-8, assay range 0.2–10 ng·mL^−1^, and 0.08 ng·mL^−1^ for TIMP-1, assay range 0.2–10 ng·mL^−1^. All samples were assayed in duplicate. The concentrations of IL-1β, MMP-8, and TIMP-1 in each sample were then corrected based on the volume of GCF.

### Subgingival plaque sampling and microbial composition analysis

In parallel to and after GCF collection, subgingival plaque samples were collected using a sterile Gracey curette from the sulcus of the distal site of targeted M2s, placed into an Eppendorf tube containing 1 000 μL of PBS, and immediately stored at −80 °C for DNA extraction. The microbial DNA was isolated as previously described.^44^ In brief, genomic DNA was isolated and purified from the subgingival plaque samples using a commercially available kit (QIAamp DNA Mini Kit, QIAGEN Sciences, USA) according to the manufacturer’s instructions. The V3–V4 hypervariable regions of the 16S rRNA gene were amplified using the 515F/608R universal primer with the barcode and subjected to high-throughput sequencing using the Illumina NovaSeq6000 platform. The raw sequencing data were processed using the pipeline tools of QIIME 1.9.1 (http://qiime.org/scripts/split_libraries_fastq.html). After trimming and filtering, high-quality sequences were aligned to the Silva 132 database (http://www.arb-sliva.de/) and clustered into OTUs using Uparse software (Uparse v7.0.1001, http://drive5.com/uparse/) at a 97% similarity level.

### Statistical analysis

Data were recorded and analyzed at the tooth level. Missing values due to loss to follow-up were deleted. Decisions about whether to use parametric or nonparametric tests were made based on the results of the Kolmogorov–Smirnow test for normal distribution. Nonparametric tests were used for intra- and intergroup comparisons (Wilcoxon signed-rank test for matched comparisons, Mann–Whitney *U* test for intergroup comparisons, and Friedman test for comparisons among multiple periods). The chi-square test and Cochran’s *Q* test were used to compare the proportions of PD5+ and BOP+. The diversity of the microbial community was determined by alpha diversity indices, including the ACE, Chao1, Shannon, and Simpson indices. The comparison of the microbial structure was conducted using PCoA based on weighted UniFrac distance. The distribution of the relative abundance of the top 35 genera was plotted using a heatmap. MetaStats analysis was used to determine the statistical significance of differences in relative abundance at different time points. The two-sided significance value was set at 0.05. For multiple testing, the *P* value was further adjusted by the Bonferroni correction. All statistical analyses were performed with QIIME 1.9.1, R 2.15.3 (R Foundation, Vienna, Austria), and SPSS (v20.0, IBM, Chicago, IL, USA).

## Conclusion

In this longitudinal study, we investigated how the periodontal condition changed in response to neighboring N-M3 removal across a 6-month period. For the first time, the present study demonstrated that N-M3 removal was associated with an improvement of the periodontal condition around neighboring teeth, including superior clinical parameters, decreased GCF inflammatory biomarkers, and reduced pathogenic microbiome distribution within subgingival plaque. Our findings provide direct evidence that N-M3s exert adverse effects on the periodontal health of adjacent M2s. Although prophylactic removal of N-M3s remains controversial, we suggest that an early medical decision should be made on N-M3s before the neighboring teeth are irretrievably damaged.

## Supplementary information

Supplementary Figures

Supplementary Table

## Data Availability

The raw sequence data are available in the NCBI Sequence Read Archive under BioProject PRJNA656079, and data that support the other findings in this study are available from the corresponding author upon reasonable request.
