# Supplementary material for: Removal of nonimpacted third molars alters the periodontal condition of their neighbors clinically, immunologically, and microbiologically
Source: Int J Oral Sci. 2021 Feb 7;13:5. doi: 10.1038/s41368-020-00108-y (PMC7867655; doi:10.1038/s41368-020-00108-y)
Supplement: Supplementary file 1 — Supplementary Figures [file 41368_2020_108_MOESM1_ESM.docx]

**Supplementary Figures**

**
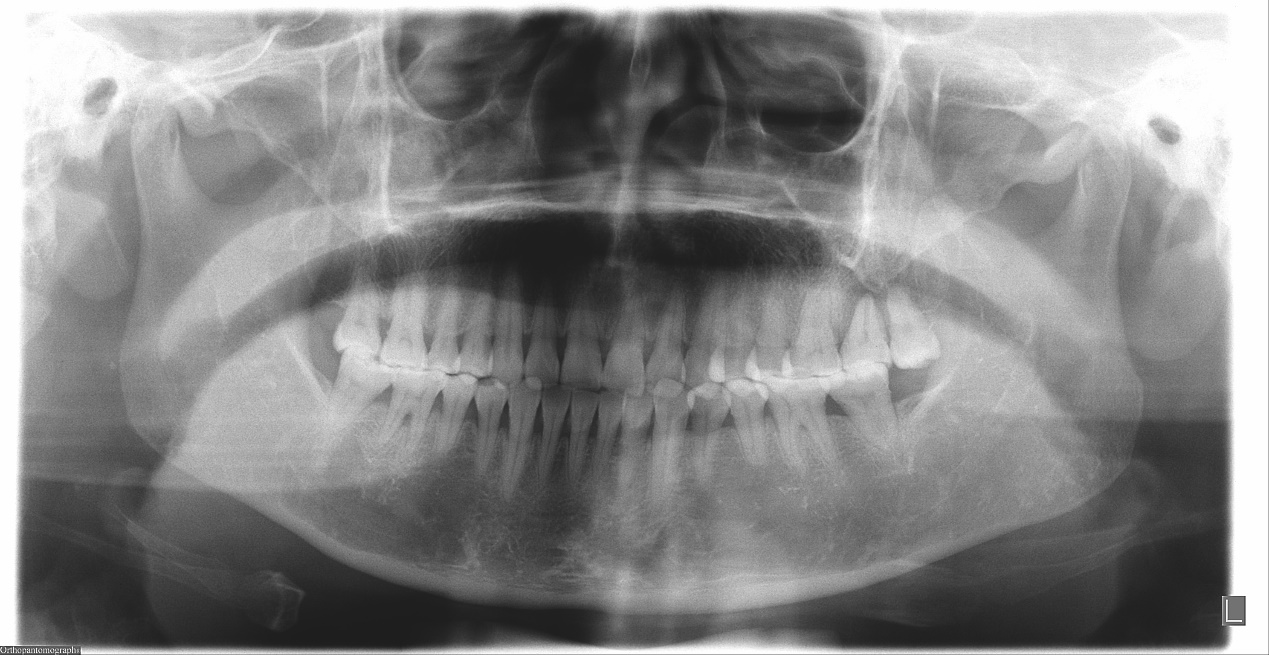
**

**Supplementary Figure** 1. Panoramic film shows the adverse effects of N-M3 on periodontal health of adjacent M2 (N-M3 located in left side of maxillary jaw)

**
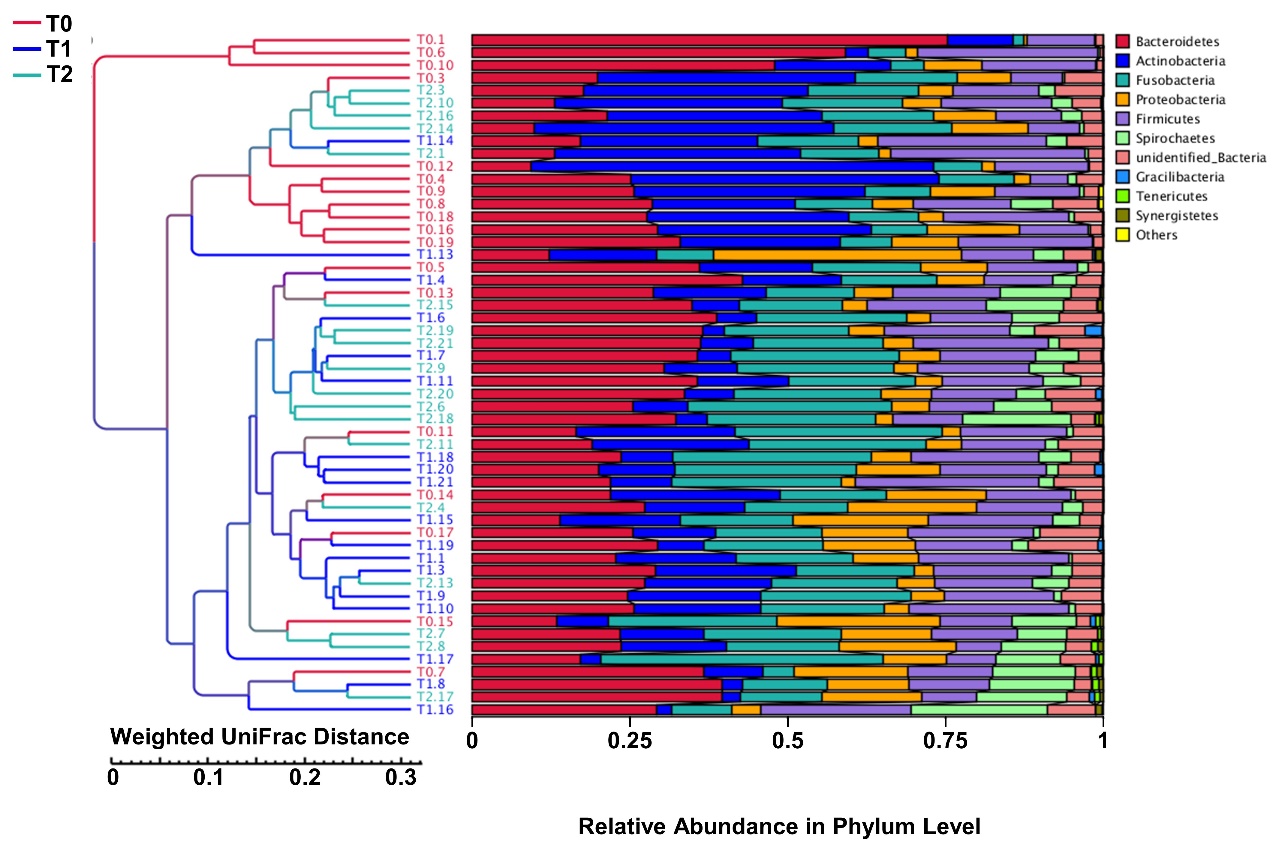
**

**Supplementary Figure** 2. UPGMA clustering analysis based on weighted UniFrac distances of each sample at the phylum level. UPGMA, unweighted pair group method with arithmetic mean; T0, before N-M3 removal; T1, 3 months post N-M3 removal; T2, 6 months post N-M3 removal.
