# Supplementary material for: Removal of nonimpacted third molars alters the periodontal condition of their neighbors clinically, immunologically, and microbiologically
Source: Int J Oral Sci. 2021 Feb 7;13:5. doi: 10.1038/s41368-020-00108-y (PMC7867655; doi:10.1038/s41368-020-00108-y)
Supplement: Supplementary file 2 — Supplementary Table [file 41368_2020_108_MOESM2_ESM.docx]

**Supplementary Table**. Spearman correlation coefficient between GCF inflammatory biomarkers (concentrations of IL-1β, MMP-8, TIMP-1 and ratio of MMP-8/TIMP-1) and the relative abundance of pathogenic microbiome (genera *unidentified Prevotellaceae* and *Streptococcus*) (*N* = 18).

|  | **IL-1β** | | | **MMP-8** | | | **TIMP-1** | | | **MMP-8/TIMP-1 ratio** | |
| --- | --- | --- | --- | --- | --- | --- | --- | --- | --- | --- | --- |
|  | ***r*** | ***p*** | ***r*** | | ***p*** | ***r*** | | ***p*** | ***r*** | | ***p*** |
| ***Unidentified Prevotellaceae*** | 0.204 | 0.140 | 0.212 | | 0.123 | 0.239 | | 0.082 | 0.133 | | 0.339 |
| ***Streptococcus*** | - 0.068 | 0.623 | 0.084 | | 0.548 | 0.118 | | 0.394 | 0.047 | | 0.733 |

**NOTES:** IL-1β, interleukin-1β; MMP-8, matrix metalloproteinase-8; TIMP-1, tissue inhibitors of matrix metalloproteinase-1

significance value was set at 0.05.
